# Current Knowledge of the Mode of Action and Immunity Mechanisms of LAB-Bacteriocins

**DOI:** 10.3390/microorganisms9102107

**Published:** 2021-10-07

**Authors:** Adrián Pérez-Ramos, Désiré Madi-Moussa, Françoise Coucheney, Djamel Drider

**Affiliations:** UMR Transfrontalière BioEcoAgro 1158, Univ. Lille, INRAE, Univ. Liège, UPJV, YNCREA, Univ. Artois, Univ. Littoral Côte d’Opale, ICV—Institut Charles Viollette, F-59000 Lille, France; adrian.perez-ramos@univ-lille.fr (A.P.-R.); desire.madimoussa.etu@univ-lille.fr (D.M.-M.); francoise.coucheney@univ-lille.fr (F.C.)

**Keywords:** lactic acid bacteria, bacteriocin, antimicrobial peptide, mode of action, immunity

## Abstract

Bacteriocins produced by lactic acid bacteria (LAB-bacteriocins) may serve as alternatives for aging antibiotics. LAB-bacteriocins can be used alone, or in some cases as potentiating agents to treat bacterial infections. This approach could meet the different calls and politics, which aim to reduce the use of traditional antibiotics and develop novel therapeutic options. Considering the clinical applications of LAB-bacteriocins as a reasonable and desirable therapeutic approach, it is therefore important to assess the advances achieved in understanding their modes of action, and the resistance mechanisms developed by the producing bacteria to their own bacteriocins. Most LAB-bacteriocins act by disturbing the cytoplasmic membrane through forming pores, or by cell wall degradation. Nevertheless, some of these peptides still have unknown modes of action, especially those that are active against Gram-negative bacteria. Regarding immunity, most bacteriocin-producing strains have an immunity mechanism involving an immunity protein and a dedicated ABC transporter system. However, these immunity mechanisms vary from one bacteriocin to another.

## 1. Introduction

Production of bacteriocins can give a bacterium a competitive advantage in its natural environment. Bacteriocins are synthesized by both Gram-negative and Gram-positive bacteria, and also by *Archaea,* using the ribosomal machinery [1]. LAB-bacteriocins are usually active against phylogenetically related bacteria [2]. Nevertheless, some of them possess a broader spectrum that includes activity against more phylogenetically distant bacteria [3,4]. In this respect it has been reported that LAB-bacteriocins such as bacteriocin OR-7 [5], bacteriocin SMXD51 [6], and E20 fraction [7] are even active against Gram-negative bacteria such as *Campylobacter jejuni* and *Escherichia coli*, including *E. coli* strains carrying *mcr-1* gene, which is responsible for colistin resistance [8]. The mode of action of LAB-bacteriocins against Gram-negative bacteria remains to be understood.

To date, a large number of bacteriocins have been isolated and characterized, which has permitted academic advances and possibilities of potential applications. Related to that, LAB-bacteriocins have been also shown to display other biological functions such as antiviral activity, inhibition of biofilm formation and anti-cancer activity [9,10]. Different classifications of bacteriocins have been proposed and include LAB-bacteriocins as well as those produced by other Gram-positive or Gram-negative bacteria [11,12,13,14,15,16].

**Figure 1:**
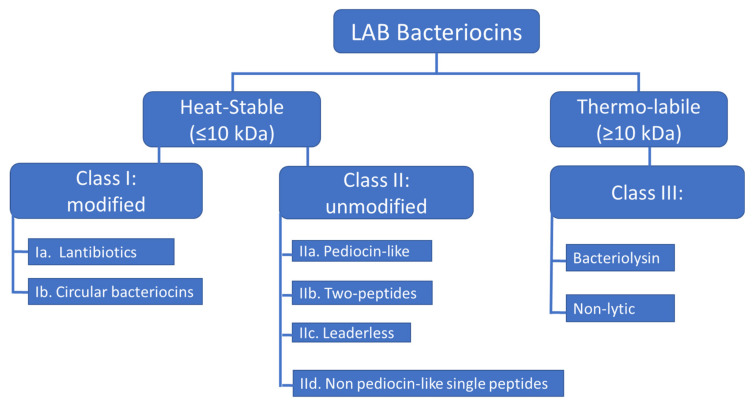
Suggested classification scheme for LAB-bacteriocins, adapted from Alvarez-Siero et al. [14].

Rebuffat and coworkers [17] have suggested classifying bacteriocins produced by Gram-negative bacteria into two main classes. The first includes high molecular mass modular proteins (30–80 kDa) termed colicins-like, whilst the second contains low molecular mass peptides (from 1 to 10 kDa) termed microcins-like. Recently, a novel classification containing bacteriocins from Gram-positive bacteria and Gram-negative bacteria has been proposed by Soltani et al. [16], and also proposes two main classes. The first contains ribosomally synthesized and post-translationally modified peptides (RiPPs) and the second encompasses unmodified peptides [16]. Importantly, there is not a common, universally accepted classification and this issue has to be considered as important by the growing scientific community dedicated to bacteriocins. In this review, we will be mostly referring to the classification proposed by Alvarez-Sieiro et al. [14], which categorizes LAB-bacteriocins into three main classes (Figure 1). Class I consists of low molecular weight bacteriocins (≤10 kD), carrying post-translational modification characterized by the presence of unusual amino acids (lanthionin), class II consists of other low molecular weight bacteriocins (≤10 kD) which do not undergo post-translational modification and class III consists of all high molecular weight bacteriocins (10 kD).

Applications of LAB-bacteriocins have been mostly limited to the food sector [18,19], and only the lantibiotic nisin (E234) and, to a lesser extent, the pediocin PA-1/AcH are currently approved and commercialized as a food preservative by the Food and Drug Administration (FDA) [20]. Further bacteriocins such as colicins and salmocins, which are produced by Gram-negative bacteria are considered by the FDA [21]. Nevertheless, safe and potent antibacterial agents, such as LAB-bacteriocins, are expected to be used in the framework of the novel approach to food preservation, named biopreservation; a method which is gaining in importance and meeting societal and industrialists’ expectations, in terms of substitution of currently used chemicals, and food shelf-life extension. In addition, LAB-bacteriocins has others potential applications such as treatment or prevention of diseases and human/animal health (Table 1).

As mentioned above, LAB-bacteriocins have been shown to display multiple biological functions [9,10]. Nonetheless, replacement of traditional antibiotics or potentiating their activity is timely, because of the increasing antimicrobial resistance in the globe and the lack, in some cases, of therapeutic alternatives. Bacteriocins, and ultimately LAB-bacteriocins may help to save us from a well-acknowledged crisis.

To protect themselves from their own bacteriocins, producing strains have developed different mechanisms to overcome the toxicity of their own bacteriocin arsenal. [12,44,45]. Genes coding for bacteriocins and those coding for immunity proteins are usually located in the same cluster and are often co-regulated.

This review discusses the mode of action and immunity mechanisms of LAB-bacteriocins and highlights recent and pertinent advances achieved in these fields. We will also show how the immunity systems, and mode of actions, can be connected. Overall, understanding advances achieved in these fields can help improve LAB-bacteriocin production rates as well as in the engineering of novel bacteriocins with potent antibacterial activity.

## 2. RiPPs, the Bacteriocins with Versatile Modes of Action

RiPPs belong to a group of antimicrobial peptides (AMP) known for their ability to undergo post-translational enzymatic modifications during their biosynthesis. As mentioned above, these AMP contain unusual amino acids and structures which play a major role in their antimicrobial activity (e.g., lanthionine, heterocycle, head-to-tail cyclization, glycosylation). They are produced by *Archaea*, as well as by bacteria and eukaryotic cells, and their classification is based on the post-translational modifications [46].

### 2.1. Class Ia: Lantibiotics

**Figure 2:**
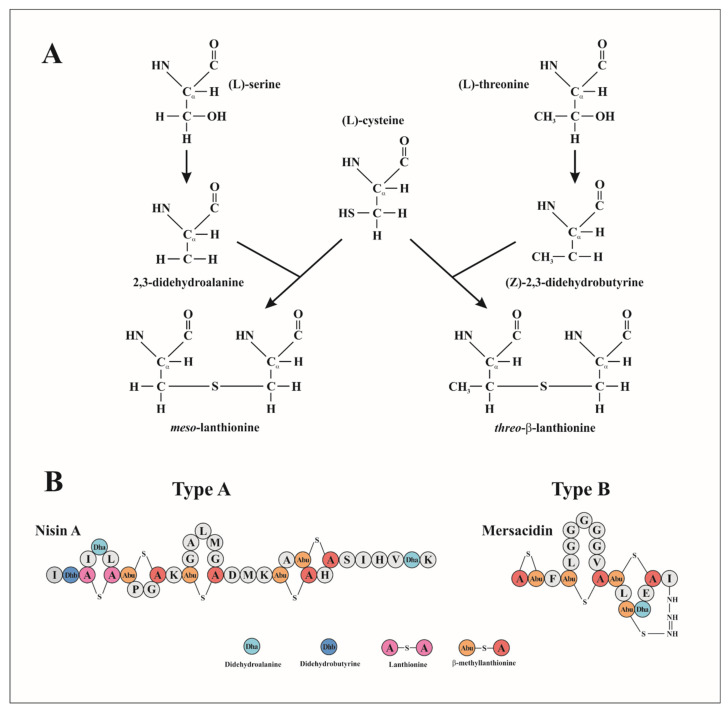
(**A**) Biosynthesis of the thioether lanthionines during lantibiotic maturation. Dha (2,3-didehydroalanine) and Dhb (2,3-didehydrobutyrine) are obtained from the dehydration of L-serine and L-threonine, respectively. Then, Dha and Dhb react with L-cysteine to form a Lan thioether resulting in lanthionine (*meso*-lanthionine) and β-methyllanthionine (*threo*-β-lantionine), respectively. Lantibiotics are divided in two types, A and B. (**B**) Molecular structure of nisin A (type A), and mersacidin (type B), where the modified amino acids are highlights in different colors. Adapted from McAuliffe et al. [44].

Lantibiotics are low molecular weight RiPPs which contain unusual dehydroamino acids such as the thioether amino acids lanthionine (Lan) and/or β-methylanthionine (MeLan) and various other modified residues, such as α,β-unsaturated amino acids 2,3-didehydroalanine (Dha) and 2,3-didehydrobutyrine (Dhb) resulting from the dehydration of serine and threonine residues, respectively. They can next form thioether β-carbon linkages with cysteines (Figure 2A) [44]. These unusual amino acids play an important role in the rigidity of the peptide and its resistance to proteolytic degradation and to heat treatment [47].

Lantibiotics are subdivided into types A and B, according to their structural and functional characteristics (Figure 2B) [48]. Type A lantibiotics, includes nisin, epidermin and subtilin are cationic and linear peptides with similarities in the arrangement of their Lan bridges. They act on the membrane of their targets by pore-forming leading to the leakage of small molecules [49]. Type B lantibiotics include duramycin, mersacidin and actagardin which are globular peptides that inhibit the synthesis of the cell wall of their targets by disrupting the enzyme function.

#### 2.1.1. Mechanism of Action of Lantibiotics

**Figure 3:**
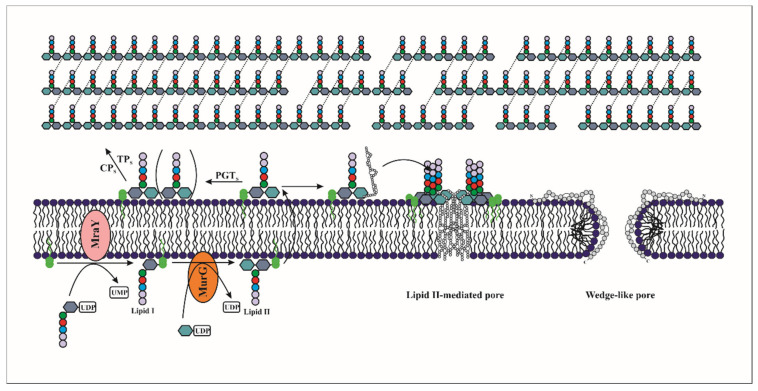
Scheme showing the mechanism of action for lantibiotic bacteriocins, mainly forming a pore into the target membrane by electrostatic interaction (wedge-like pores) or by binding to lipid II. Lipid II is formed in two steps: (i) the UDP-N-acetylmuramyl-pentapeptide is linked to the undecaprenyl phosphate by the enzyme MraY to obtain the lipid I; (ii) then, a unit of N-acetylglucosamine is linked to the N-acetylmuramyl by the enzyme MurG to obtain the final lipid II. After its formation, the lipid II is translocated across the membrane to the outer side, and oligomerized to form the cell wall. The cationic type-A lantibiotics (like nisin, depicted in the figure) can form a target-mediated pore using lipid II as a docking molecule. In addition, the hijacking of lipid II molecules for the formation of pores in the membrane can destabilise the formation of the wall, thereby weakening it.

Lantibiotics act by disrupting the cell wall biosynthesis of their target bacteria, or/and by forming a pore in their membranes. Of note, lantibiotics have been shown to bind to lipid II and interfere with the transport of peptidoglycan subunits from the cytoplasm into the cell wall and consequently inhibit cell wall formation. They can also use lipid II as a docking molecule to initiate pore formation and lead to rapid cell death (Figure 3) [45,50,51].

(A) Pore formation

Lantibiotics can interact with the cytoplasmic membrane of their targets and form pores [52]. These peptides of generally cationic nature can cause the dissipation of the proton motive force (PMF), through the formation of pores in the cytoplasmic membrane [44]. The PMF results from the electrochemical gradient of protons across the cytoplasmic membrane, it is constituted of an electrical component, the membrane potential (ΔΨ), and a chemical component, the pH gradient (ΔpH) [53,54]. The disruption of the PMF induced by the bacteriocin, leads to cell death by stopping the reactions requiring energy.

Type A lantibiotics such as nisin, Pep5, subtilin, lacticin 3147 and streptococcin FF22, form pores in the membranes of the target cells in a process of several steps, starting from the interaction of the bacteriocin with the membrane to its insertion in the phospholipidic bilayer that causes the release of low molecular weight intracellular compounds such as amino acids, ions and ATP [55,56,57]. These cationic peptides interact with the anionic surface of the cytoplasmic membrane. The C-terminal region containing the majority of positively charged residues binds to the head group of anionic phospholipids in the cytoplasmic membrane by electrostatic interactions [58]. The insertion of lantibiotics into the cytoplasmic membrane is generally mediated by the hydrophobic residues of their N-terminal region (Figure 3) [59].

Two different models of pore formation by lantibiotics have been proposed: "barrel-stave” and “wedge” models [60]. In the barrel-stave model, the lantibiotic monomers bind by electrostatic interaction to the outer leaflet in an orientation parallel to the membrane surface. Thus, a water-filled pore is formed following the interaction of the hydrophobic lipid core of the membrane with the non-polar side chains of the bacteriocin. The size and stability of the barrel-stave pore depends on the number of peptides involved [56,60]. However, in the wedge model, a local deformation of the membrane is observed due to the interaction between cationic bacteriocins and the head groups of the phospholipids which are anionic allowing the insertion of the hydrophobic residues of the bacteriocin inside the membrane (Figure 3) [61].

The formation of the pores in the cytoplasmic membrane is accompanied by an increase of the free energy [62]. The electrical transmembrane potential (Δψ), generated by bacterial cells in the metabolic phase is considered as the main driving force for activity. Experiments on black lipid membranes (which are lipid membrane models used for the investigation of biomembrane properties) indicated that when the trans-negative electrical potential is reached the nisin formed pores, the similar orientation is obtained in the bacterial membrane. [55,63]. Similarly, the pore formation can be impacted by pH. Garcera et al. [64] reported that at high pH, the nisin monomers aggregate outside the membrane, which significantly reduces its activity.

Pore formation can be enhanced by the association of lantibiotics with the polyisoprenoid-linked cell wall precursor, lipid II. Its structure is highly conserved in bacteria, and consists of a pentapeptide bound to the disaccharide unit N-acetylmuramyl–N-acetylglucosamine (MurNAc-GlcNAc), as well as a bactoprenol carrier lipid (C55-P), which is linked to the disaccharide unit via a pyrophosphate bridge [65]. Nisin and epidermin, use lipid II as an anchor molecule to bind specifically to the cytoplasmic membrane. Moreover, the presence of lipid II causes pore stabilization and enhances the sensitivity of the target membrane to nisin (Figure 3) [51,66]. In vitro experiments have shown that the presence of lipid II significantly increases the activity of nisin (from a micromolar to nanomolar concentration) [67]. NMR analysis of the interaction of nisin and lipid II has shown a network of five intermolecular hydrogen bonds between the peptide backbone of nisin and the pyrophosphate domain [68]. Similarly, other studies revealed the presence of a conserved motif in the N-terminal region that would be involved in the binding to lipid II and this motif is present in some type A(I) lantibiotics such as subtilin, epidermin, gallidermin and mutacin [69]. In addition to forming highly specific pores, nisin can also inhibit cell wall synthesis through its binding to lipid II, as we detail in the next section. The combination of two mechanisms of action increases markedly the bactericidal activity, being effective even down to the nanomolar range [51].

(B) Inhibition of peptidoglycan biosynthesis mediated by Lipid II interaction

Lantibiotics such as gallidermin that bind to lipid II kill bacteria without permeabilizing their membranes, indicates another lipid II-mediated mode of action [70]. Indeed, the type-B lantibiotics mersacidin and actagardin, whose structures are completely different from nisin, impede the transglycosylation reaction during biosynthesis of the peptidoglycan by forming a complex with lipid II (Figure 3). Of note, it was established in the *Staphylococcus simulans* 22 strain, that mersacidin blocks the incorporation of glucose and D-alanine into the cell wall, thus stopping its biosynthesis and leading to cell death [60]. In vitro studies performed on membranes capable of synthesizing peptidoglycan showed that inhibition of peptidoglycan synthesis by mersacidin was dependent on the availability of lipid II [60,71].

The highly conserved cyclic structure in both lantibiotics could be involved in the antibacterial activity. Comparison of mersacidin with other similar lantibiotics suggests the presence of a conserved motif in the N-terminal region that is involved in lipid II binding [72]. The binding site of mersacidin and actagardin on lipid II is not the same as that of nisin, they interact mainly with the disaccharide-pyrophosphate fraction of lipid II [71,73].

(C) Other mechanisms of action

Bierbaum and Sahl [74] showed that both nisin and Pep5 can induce autolysis in staphylococci. They suggested that nisin and Pep5 bind to lipoteichoic and teichoic acids and displace the enzymes N-acetylmuramoyl-L-alanine amidase and N acetylglucosaminidase, which normally interact with teichoic acids, leading to activation of the enzymes involved in cellular lysis. Some lantibiotics can act also inhibit the germination of *Bacillus* spores. It has been reported that the mechanism of action of subtilin in *Bacillus cereus* T vegetative cells, or spores can follow different molecular pathways, and also it was observed that the Dha residues at position 5 of the molecule were essential in the inhibition of spores [75].

#### 2.1.2. Lantibiotic Immunity

This system consists of an immunity protein (LanI) or an ABC transporter (composed of 2 or 3 LanEF(G) subunits). Both, LanI and LanEF(G) can act independently or interact together to confer autoimmunity. Of note, in some cases, an accessory protein called LanH is involved in this overall self-protection network [44].

The regulation of biosynthesis of many lantibiotics is controlled by a two-component signal transduction system, the histidine kinase (LanK) and the response regulator (LanR). The pre-lantibiotic activates its biosynthesis and other proteins involved in transport and immunity, by acting as a pheromone peptide through a quorum sensing and allowing autophosphorylation of the histidine kinase. LanK transfers its phosphate group onto LanR, which activates the transcription of genes belonging to bacteriocin clusters [14,44,76]. In certain cases there is an independent promoter P*_nisI_*, which provides the cell with a basal level of immunity through a weak constitutive activity, thereby protecting it during its first encounter with nisin [77].

**Figure 4:**
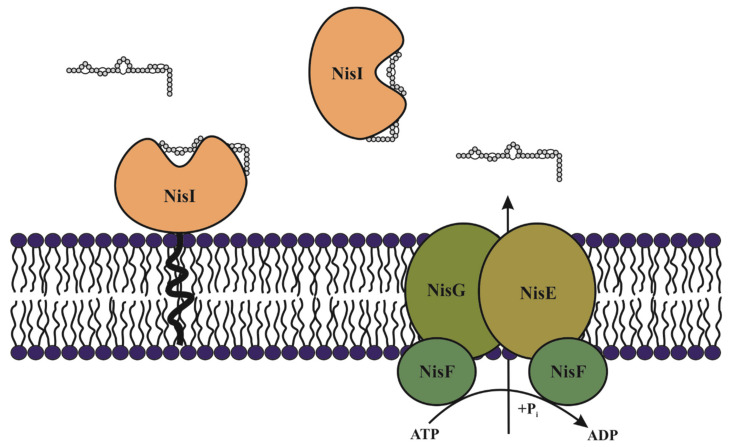
Mechanisms of immunity in the nisin producing *L. lactis* to protect itself from the action of the nisin. The lipoprotein NisI can be anchored to the membrane or released into the medium, and in both cases, it is able to bind to nisin, inhibiting pore formation. The ABC transporter NisEFG expels nisin from the membrane, thus avoiding pore formation.

Nevertheless, it has been established that *Lactococcus lactis* nisin producing strains are able to prevent the deleterious effects of its own nisin by using two distinct cooperative mechanisms. The first is related to expression of the specific NisI immunity protein, which is a lipoprotein able to bind to nisin, and thereby prevents it from accessing its predicted target lipid II (Figure 4) [78]. The second mechanism involves the ATP-binding cassette transporter NisFEG, which expels nisin outside of the membrane prior to pore formation [79,80]. These mechanisms involving NisI and NisFEG were among the first reported, since full immunity was only observed in the presence of both proteins (Figure 4) [81]. Moreover, other studies also showed the influence of both systems, and reported immunity levels of 5–20% for each mechanism [81,82]. A modification of the lipid profile allowed the anchoring of NisI extracellular side of the membrane, but a lipid-free form of the NisI also exists, secreted into the surrounding medium, and both forms were shown to bind nisin [79,83]. NisFEG seemed to recognize the C-terminal domain of nisin since deletion of the last six amino acids and last ring lowered the degree of immunity displayed by NisFEG [84]. Nevertheless, knowledge of the physiology and regulation of genes involved in this self-immunity is limited. Involvement of other genes in nisin immunity through direct or indirect interactions has been reported by Zhu et al. [85]. These authors established that the Mu-transposon occurring within the chromosomal *feuD* gene in *L. lactis* N8 provided a derivative strain *L. lactis* L58 with an enhanced nisin immunity system. Interestingly, the gene cluster *feuABCD* encoding for an ATPase of the ABC-type cobalamin/Fe3+ siderophores transporter FeuABCD including FeuD has been observed in many bacterial species [86]. This result suggested that FeuD was a component of transporter FeuABCD.

Similarly, the Gram-positive *Bacillus subtilis* has similar mechanisms to prevent toxicity of subtilin. This occurs through expression of the SpaI immunity protein and ABC transporters SpaFEG [87]. Recently, expression of SpaI and NisI in a subtilin nonproducing strain, permitted responsive resistance to both lantibiotics [88]. NMR resolution of NisI lipoprotein revealed two structurally similar domains, namely N-and C-terminal domains, which are homologous to SpaI [78,89]. The N-domain interacts with the membrane, whereas the C-terminal domain plays a role in the nisin recognition [78,89]. The N-terminal domain (1-111 residues) is linked to the C-terminal domain (112-226 residues) through a flexible linker (112-119 residues) [90]. Recently, it was reported that the inter-domain of NisI could form a deep cleft and a groove which is recognized as a binding site for nisin, among other molecules [91].

Whilst knowledge on NisI and NisFEG is increasing, it is important to know whether these mechanisms are unique or interchangeable, as recently reported for subtilin [88].

Although subtilin is the lantibiotic sharing the closest similarity with nisin (i.e., 63% sequence identity), the SpaI-NisI fusion protein, expressed in *L. lactis*, where the corresponding 21 amino acid fragment of the subtilin immunity peptide, SpaI, has been replaced by that of NisI conferred immunity to nisin, confirming that the specific protective capabilities against nisin are located at the C-terminus of the LanI proteins [78].

In contrast, the immunity protein PepI that protects the *Staphylococcus epidermidis* strain from its own bacteriocin Pep5 has a different mode of action than nisin. Indeed, this protein, containing an apolar N-terminal and a positively charged hydrophilic C-terminal region, accumulates at the interface of the membrane-cell wall. Experiments aimed at disrupting the N-terminal or the C-terminal region of PepI have shown that the N-terminal region is required for the transport of PepI, and the C-terminal region is important to confer the immunity phenotype [92].

### 2.2. Class Ib or Circular Bacteriocins

Circular bacteriocins are peptides post- translationally circularized by a head-to-tail peptide bond. This is particularly important to ensure high resistance to temperature and pH variations and protection against many proteases. Circular bacteriocins are produced by Gram-positive bacteria mainly from the *Firmicutes phylum*. They are classified into two groups based on their sequence homologies, and their physico-chemical characteristics. Group I contains circular cationic bacteriocins with high isoelectric point, whereas group II contains circular and highly hydrophobic bacteriocins with more acidic residues, and low isoelectric points [93].

#### 2.2.1. Mode of Action of Circular Bacteriocins

The biochemical and biological characteristics of many of these bacteriocins are well-known, especially enterocin AS-48 (AS-48), which has been widely studied and reviewed [93,94,95,96,97]. AS-48 was the first circular bacteriocin to be described. It is produced by *Enterococcus faecalis* subsp. *liquefaciens* strain S-48 [98], and is considered as the prototype of this family. Its active form is a cationic circular peptide of 70 residues and a molecular weight of 7.1 kDa, folding in an all-α-helix with five helical regions, where the recircularization of the backbone occurs in the middle of helix a5 [94]. Its mechanism of action has been thoroughly investigated, but there are still issues to be clarified. AS-48 has the cytoplasmatic membrane as its target, where it destabilizes the membrane leading to the dissipation of the proton motive force and cell death. Crystallographic studies showed that the protein dimerizes and undergoes a transition from a dimer water-soluble form (DF-I) to a dimer membrane-bound state (DF-II) [99]. Dimer formation in DF-I is mediated by hydrophobic interactions, whereas in DF-II hydrophilic contacts occur between protomers, a hydrophobic surface is exposed to the hydrophobic core of the membrane and a polar surface remains in contact with the solvent. The experimental data suggest that AS-48 in the DF-I form reaches the membrane by an electrostatic approach. The acidic pH in the membrane surface would protonate the glutamic acids allowing the interactions with the phospholipids and leading the destabilization of the DF-I form. This situation would promote a rearrangement of the dimer provoking its insertion into the membrane and adopting the DF-II form [100]. Simulations of membrane permeation suggested that the protein inserted could organize itself to generate stable pores [101,102], however there are no experiments to support this. In addition, there is no evidence of the requirement for a membrane receptor for the bactericidal action of AS-48. Nevertheless, it was reported that the bactericidal activity of garvicin ML against *L. lactis* increased in the presence of the maltose ABC transporter, suggesting that this membrane protein could be involve in the antimicrobial activity [103].

N-terminal regions of circular bacteriocins carry several aromatic and/or hydrophobic amino acids suggesting that these residues play a key role in the biosynthesis and probably the antimicrobial activity [96]. Recently, a structural alignment study of different circular bacteriocins revealed the involvement of charged and aromatic conserved amino acids in antimicrobial activity [104]. Indeed, the authors showed that the substitution of phenylalanine and tryptophan residues by alanine by site-directed mutagenesis of the bacteriocin plantacyclin B21AG produced by *Lactiplantibacillus plantarum* B21 resulted in a strong reduction of the activity compared to the wild type.

#### 2.2.2. Immunity of Circular Bacteriocins

The genetic determinants for the synthesis, transport and immunity of circular bacteriocins are clustered in one or several operons, which are located on plasmids or on the chromosome. A cluster of ten genes, *as-48ABCC_1_DD_1_EFGH*, located on the 68 kbp pheromone-response plasmid pMB2, has been described for AS-48 production [105]. Northern blotting studies demonstrated that they work as two coordinated operons, *as-48ABC* and *as-48C_1_DD_1_EFGH* respectively [106]. Both are essential for bacteriocin production and immunity. Recently, the promoters involved in the expression of both operons have been investigated and, also, an additional promoter involve in *as-48D_1_EFGH* expression was confirmed [107]. D_1_ was first described as the immunity protein due to it conferring, by itself, a certain degree of bacteriocin resistance [108]. However, it has subsequently been reported that protein C plays an auxiliary role in self-protection [109]. Both are integral membrane proteins with two (D_1_) or four (C) putative transmembrane domains. Nevertheless, the mechanism of action by which they exert their immune function has not been elucidated, although it is speculated that they may interact with the putative pores formed by the bacteriocin. The *as-48EFGH* genes encode a secondary ABC transporter also involved in self-protection. It conferred higher resistance when AS-48 was added into the medium, therefore it was postulated that this ABC transporter enhances the secretion of bacteriocin by expelling it from the membrane [106]. But this transporter cannot export the newly synthetized bacteriocin; that is carried out only by the main transporter formed by the C_1_D proteins. Then, the combined expression of the complementary ABC transporter and the membrane proteins C and D_1_ are required to render a full immunity against AS-48.

A similar immunity system was reported for circularin A, where *cirGHI* genes encode a multicomponent ABC transporter identical to that of AS-48, and CirE a transmembrane protein which would act like As-48D_1_ [110]. A secondary ABC transporter was also reported for carnocyclin A [111] and for garvicin ML [112], thus it could be presumed to have a similar immunity system to AS-48. Other small transmembrane proteins were identified as immunity proteins in other circular bacteriocins, such as BviE in butyrivibriocin AR10 [113], or UblE in uberolysin [114]. In the latter, the secretory transporter UblCD was reported to confer low levels of immunity.

## 3. Class II, A Group with Not Only Pore Forming Bacteriocins

Class II constitutes a relatively heterogeneous group of low molecular weight bacteriocins (less than 10 kDa). They are characteristically thermostable and unmodified peptides. Although they are most often synthesized as pre-peptides whose N-terminal leader sequence is cleaved, the maturation of class II bacteriocins do not require post-translational modification enzyme except a leader peptidase and/or a transporter. This class includes a wide variety of structures, which has led to the creation of several subclasses.

### 3.1. Class IIa Bacteriocins, or Pediocin-Like Bacteriocins (PLBs)

Class IIa bacteriocins, known as pediocin-like bacteriocins, are linear polypeptides of up to 60 amino-acids with potent activity against *Listeria*, particularly *Listeria monocytogenes*, able to kill target bacteria at concentrations in the sub-nanomolar range [45,115]. 

These linear polypeptides have at least two residues of cysteine in the well conserved hydrophilic and charged N-terminal domain [12,116]. A second disulphide bond linkage can exist in the cases of sakacin G, plantaricin 423, pediocin PA-1/AcH, divercin V41, and enterocin A. This second disulfide bridge is expected to stabilize the 3D structure of the *C*-terminal domain, and correlate with spectrum of activity. Of note, the N-terminal domain of class IIa bacteriocins is recognized by the presence of the “pediocin box”, a sequence of 5 amino acids (YGNGV). Nevertheless, in some cases, this pediocin box can be altered by the replacement of the valine residue by lysine [117]. Finally, this pediocin box is included in the conserved *N*-terminal domain YGNGVxCxK/NxxC (where X is any amino acid) [12].

#### 3.1.1. Mode of Action of Class IIa Bacteriocins

These bacteriocins acts on the target membranes forming pores that result in the permeabilization of the membranes and cell death. Initially, it was thought that positively charged residues of the C-terminal domain were sufficient for bacteriocin interaction with the membrane, through the anionic phospholipids [12]. The first studies on Pediocin PA-1 showed the ability of this bacteriocin to permeabilize lipidic vesicles, but the authors thought this effect occurred with higher concentrations of bacteriocin than necessary [118,119,120]. Thus, they proposed that a docking molecule is needed for a full efficiency of the process. Moreover, Fimland et al. [121], showed that the presence of the fragment derived from pediocin PA-1, peptide 15-mer, inhibited the bactericidal activity of pediocin PA-1, by interfering specifically with the pediocin–target cell interaction. This docking molecule proved to be the membrane-associated EIICD components for the mannose PTS system, which interact directly with the bacteriocin [122]. However, although both components are necessary for the receptor function, it is an extracellular loop in the N-terminal region of the IIC component that provides the specificity in recognition [123]. In addition, the mannose PTS system is clustered into three groups, and only members of group I are recognized by class IIa bacteriocins. All this causes the reduced spectrum of action of these bacteriocins, restricted basically to bacteria phylogenetically related to the producer strains.

**Figure 5:**
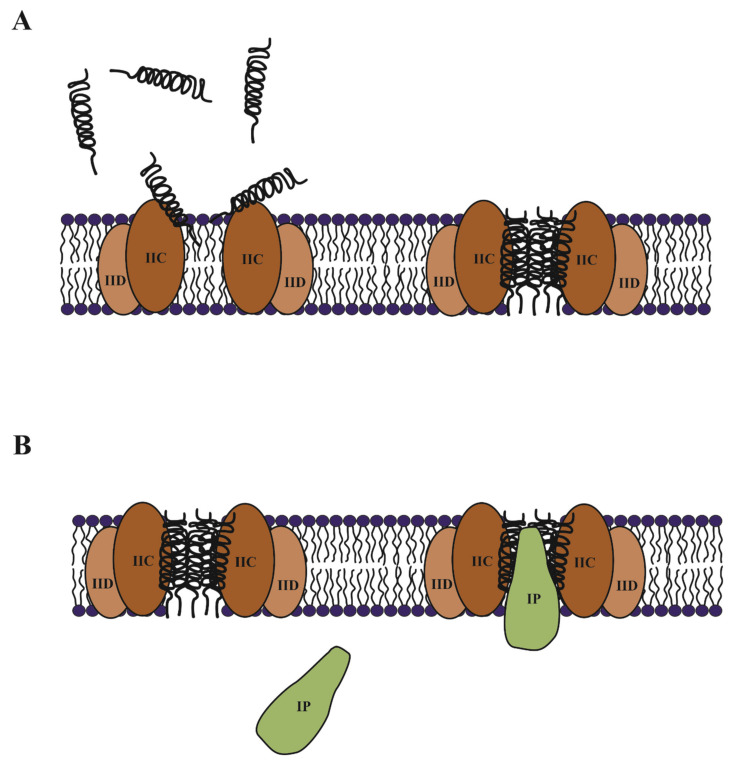
Model of the mechanism of action and immunity for the class IIa bacteriocin, based on the suggestions from enterocin CRL35 [125]. (**A**) The bacteriocin reaches the membrane via interaction with EIIC protein of maltose PTS transporter (EIIC and EIID components). Then, the peptides organize themselves to form the pore. (**B**) The immunity protein (IP) is a soluble cytosolic protein, which interacts with the complex bacteriocin-receptor plugging the formed pore, avoiding cell death.

The discovery of the interaction of pediocin-like bacteriocins with the IICD components led to the development of two possible models for their mechanism of action: 1) IICD are mere mediators to bring the bacteriocin to the membrane and thus favors its insertion, oligomerization and pore formation; 2) the binding of the bacteriocin to the membrane proteins causes a conformational change in them that leads to the permanent opening of the PTS channel [124]. Although the controversy has existed, recent studies conducted with a suicide probe have elucidated the issue. The studies were carried out in *E. coli* harboring a fused gene of *etpM* and *ent35*. Thus, the suicide probe is a chimeric peptide of the membrane protein EtpM and the class IIa enterocin CRL35 [125]. Enterocin CRL35 is not able to cross the outer membrane of *E. coli*, which is therefore resistant to the bactericidal activity of this peptide. However, the fused peptide EtpM-Ent35 is translocated to the inner membrane, producing membrane permeability and cell death, without the presence of its specific receptor. These results showed how class IIa bacteriocins need their receptor to reach the membrane and insert in to form the pore (Figure 5A).

#### 3.1.2. Immunity of Class IIa Bacteriocins

It is still unclear how the immune protein exerts its protective effect. As already mentioned, the immune protein acts from inside the cell and is not capable of interacting with the bacterial membrane by itself [12]. Nevertheless, somehow the immune protein must interact with the bacteriocin and/or its specific receptor. Diep et al. [122] demonstrated the formation of a strong tertiary complex involving a bacteriocin, the immune protein and the mannose PTS system. This complex was formed only when bacteriocin was present in the extracellular medium. Therefore, the bacteriocin–receptor interaction should occur for the coupling of the immunity protein, probably blocking the channel and thus preventing cell death. Recently, direct interaction of the immune protein with the specific receptor has been reported. Complementation assays with the immune protein PedB and the IICD components of different species, showed the existence of a recognition specificity, so the receptor protein could influence the functionality of the immunity protein [126]. However, once again the use of the suicide probe EtpM-Ent35 concomitantly expressed with the gene *munC* showed that the immunity protein MunC was able to avoid the deleterious effect of the enterocin CRL35 without the presence of the specific receptor [125]. This result suggests the direct bacteriocin–immunity protein interaction, where the role of the immunity protein might be blocking the pore formed by the cognate bacteriocin (Figure 5B).

### 3.2. Class IIb Bacteriocins, or Two-Peptide Bacteriocins

These bacteriocins are composed of two different peptides (usually named α- and β-peptide) which exhibit a high antimicrobial activity at pico- to nano-molar concentration, only when they are combined in equal ratio. In addition, the combination of one of the two peptides with the complementary peptide from another homologous bacteriocin can render a similar activity. This has been reported in lactococcin G, when one of the peptides was combined with the complementary peptide from lactococcin Q or enterocin 1071 [127].

All peptides from this bacteriocin class contain inside their helical domains at least one GxxxG-motif, except for plantaricin Sβ and plantaricin NC8β which contain the GxxxG-like motifs AxxxA- and SxxxS-motif, respectively [127]. These motifs are known to mediate helix–helix interactions in membrane proteins [128].

#### 3.2.1. Mode of Action of Class IIb Bacteriocins

These bacteriocins act on the membranes of their targets by rendering them permeable to certain cations such as Na+, K+, Li+ or H+. This characteristic of mediating the transport of specific cations indicates that the bacteriocins form relatively sophisticated pores [127]. Furthermore, this membrane permeability is related to low concentrations of peptides, which is consistent with pore formation, but not with a detergent-like disruption of the membranes. The narrow spectrum of these bacteriocins has suggested the involvement of a receptor for their action, as described for pediocin-like bacteriocins. Recently, the undecaprenyl-pyrophosphate phosphatase, which is involved in peptidoglycan synthesis, has been described as the receptor for lactococcin G [129]. Furthermore, other membrane-associated proteins have been identified as the receptor for two-peptide bacteriocin such as plantaricin JK [130] and plantaricin EF [39].

#### 3.2.2. Immunity of Class IIb Bacteriocins

Regarding the immunity system, it has been described as being similar to those of pediocin-like bacteriocins, where there could be a direct interaction between the bacteriocin and the immunity protein, or an indirect interaction through its membrane receptor [127]. Nevertheless, little research has been done on these type of immunity proteins and their mode of action is unknown. LagC is the immunity protein for lactococcin G. Mutational studies revealed that LagC recognizes its own bacteriocin by the N-terminal part of lactococcin Gα (residues 1 to 13) and the C-terminal part of lactococcin Gβ (residues 14 to 24) [130]. In addition, it has been reported some degrees of cross-immunity between lactococcin G and its immunity protein LagC with the homologous enterocin 1071 [130], lactococcin Q [131] and their immunity proteins, EntI and LaqC, respectively. Moreover, it was observed that the protective activity of the LagC and EntI proteins depended on the composition of the target cell membranes, suggesting the interaction of these immunity proteins with their cognate bacteriocin receptors [130]. On the other hand, some immunity proteins such as SkkI, PlnI and PlnL, involved in self-protection against their cognate bacteriocins sakacin 23K, plantaricin EF and plantaricin JK, respectively, have shown homology to the Abi family of proteins, putative membrane-bound metalloproteases [132]. Thus, it has been suggested that these immunity proteins degrade their cognate bacteriocins by proteolysis.

All the immunity proteins for two-peptide bacteriocins have been describe as membrane-associated proteins, containing at least one putative transmembrane domain. Recently, experimental procedures corroborated the membrane-associated nature of LagC and LciM, immunity proteins for lactococcin G and lactococcin MN, respectively [133]. Both proteins contain four transmembrane domains, but differ in orientation across the membrane, length and sequence. Additionally, CbnZ has also been identified as the immunity protein for carnobacteriocin XY. CbnZ contains only one transmembrane domain, and it is the smallest (42 amino acids) immunity protein described for this kind of bacteriocin up until now. Two-peptide bacteriocin immunity proteins are composed of proteins with a great structural variety [133].

### 3.3. Class IIc, Leaderless Bacteriocins

Bacteriocins are usually synthesized in an inactive form, with a leader peptide sequence that serve to guide it in its modification and secretion process. However, there is a group of bacteriocins that do not follow this scheme and do not undergo any translational modification and remarkably lack this leader sequence. These bacteriocins are thought to be synthesized in their active form, which raises questions about how the cell manages its own protection until the excretion of the peptide to the extracellular medium [97]. This group is very heterogeneous, especially regarding the number of peptides that compose the final bactericidal action, being able to be single-peptide, two-, three- or four-peptides bacteriocins. Much remains to be elucidated regarding the mechanism of action and the immune system of this group of bacteriocins.

#### 3.3.1. Mechanism of Action of Leaderless Bacteriocins

Regarding the mode of action, two mechanisms have been described. Lacticin Q binds to the target membranes by electrostatic interactions which provokes a rapid integration into the phospholipid bilayer. A large toroidal pore is formed leading to leakage of large intracellular components that causes cell death [134]. On the other hand, aureocin A53 has been shown to interact with the target membranes, leading to cell death, but without pore formation [135]. In addition, leaderless bacteriocin do not seem to require a receptor molecule to exert their antimicrobial effects. Nevertheless, the leaderless bacteriocin LsbB has been reported to interact with the zinc-dependent membrane metallopeptidase YvjB [136].

#### 3.3.2. Immunity of Leaderless Bacteriocins

The immunity systems of this heterogeneous group of bacteriocins are poorly understood. In most cases, only possible candidate proteins that might be involved have been identified. In the case of aureocin A53, an ABC transporter has been demonstrated to be involved in self-protection, but for full immunity at least one more protein is necessary [137]. Two co-transcribed proteins, AucIA and AucIB, from *orf10-11* could be involved. A mutant carrying both proteins and the ABC transporter resulted in higher self-protection than the wild-type strain. The authors could not exclude either AucIA or AucIB from the involvement in immunity. Nevertheless, AucIA shares certain characteristics with other described immunity proteins, such as being a cationic protein and being associated to the membrane by three putative transmembrane regions [137]. In aureocin A70, a four-peptide leaderless bacteriocin, an ABC transporter was described that is involved in bacteriocin externalization but not in self-protection. The immunity is given by the AurI protein, that is AucIA for aureocin A53, which is a cationic transmembrane protein [138]. The well-known lacticin Q gene is adjacent to the structural gene in an operon of five genes, *orf3-7*, involved in transport and immunity [139]. Overexpression of the structural gene of lacticin Q in *L. lactis* NZ9000 caused an intracellular toxicity that was suppressed by the co-expression of the *orf3*-7 genes. The decrease in toxicity is due to the secretion of the produced bacteriocin into the extracellular medium and by the presence of an auto-immunity mechanism. Orf6-7 form a transporter for the bacteriocin, whereas Orf3-5 are all membrane proteins, of unknown function, that could be involved in the immunity process [139].

### 3.4. Class IId Bacteriocins, and Other Non-Pediocin-Like Single-Peptide Bacteriocins

Class IId consists of a group of heterogeneous bacteriocins whose main characteristic is that they are linear single-peptides that do not have motifs common to pediocin-like peptides. Examples include lactococcin A, lactococcin 972 and enterocin B [14].

Lactococcin A is a bacteriocin produced by *L. lactis* subsp. *cremoris* LMG 213 that has activity against strains that are phylogenetically close to the producing strain. It is produced as a 75 amino acid precursor with a 21 amino acid N-terminal extension. The genes involved in the biosynthesis of lactococcin A, including the structural gene (*lcnA*), the immunity gene (*lciA*) and two genes (*lcnC, lcnD*) that encode the dedicated ABC transport system and its accessory protein, are carried on a plasmid of about 55 kb [140].

In vitro experiments performed with purified lactococcin A showed that this bacteriocin could increase the permeability of the cytoplasmic membrane of the target cells by pore formation and also cause depolarization of the membrane potential. Similarly, it has been suggested that the specificity of lactococcin A may be mediated by a receptor protein associated with the cytoplasmic membrane [52]. Diep et al. [122] demonstrated that this specific receptor uses components of the mannose phosphotransferase system (man-PTS).

Some class IId bacteriocins have a mode of action other than pore formation. This is the case for lactococcin 972 (Lcn972), a bacteriocin produced by *L. lactis* IPLA972 that inhibits septum biosynthesis in the target strain by binding to the cell wall precursor, lipid II. Because it lacks any lanthionine ring or even cysteines that could form intramolecular bridges, it has been suggested that Lcn972 recognizes another motif in lipid II other than the pyrophosphate cage that is recognized by lantibiotics [141].

In order to protect itself against its own bacteriocin, the *L. lactis* strain produces an immunity protein called LciA possessing an α-helix structure. Venema et al. [142] proposed a model of immunity mode of action in which the LciA immunity protein interacts with the man-PTS receptor of the *L. lactis* strain, thereby preventing pore formation by the bacteriocin.

## 4. Class III, “Wall Breaker” Bacteriocins

Class III includes all the thermolabile bacteriocins with a molecular weight above 10 kDa. Generally, they consist of several domains which can have different functions, such as translocation, receptor binding or lethal activity [14,45].

Enterolysin A is a class III bacteriocin produced by *E. faecalis* LMG 2333. It is active against *Enterococcus*, *Pediococcus*, *Lactococcus* and *Lactobacillus* [143]. Of note, this bacteriocin is produced as a pre-protein of 343 amino acids with a 27 amino acid sec-dependent signal peptide, followed by a sequence corresponding to the N-terminal region of the mature protein. Sequence analysis of enterolysin A revealed the presence of two distinct domains, an N-terminal catalytic domain and a C-terminal substrate recognition domain. Moreover, the N-terminal region presents homologies with sequences of catalytic domains of different cell wall degradation proteins with a modular structure, such as ALE-1, and LytM which are both endopeptidases belonging to the M37 protease family [143,144].

### 4.1. Mode of Action of Class III Bacteriocins

Enterolysin A-like, lysostaphin, zoocin and millericin B are known to be endopeptidases that act at the bacterial cell wall level. As a reminder, the cell wall of Gram-positive bacteria is composed of alternate units of N-acetyl glucosamine (NAG) and N-acetyl muramic acid (NAM) linked by β -1→ 4 bonds. The D-lactoyl group of each NAM residue is replaced with a peptide chain, containing most often L-Ala-D-Glu-L-Lys-D-Ala which is called the stem peptide. Adjacent glycan strands are connected by cross-linking peptide chains between two peptide side chains. The lengths of these cross-linking chains, also called interpeptide bridges or cross-bridge, are very different between *Staphylococcus aureus* to *Enterococcus faecium* despite the similarities in other parts of peptidoglycan [145,146].

**Figure 6:**
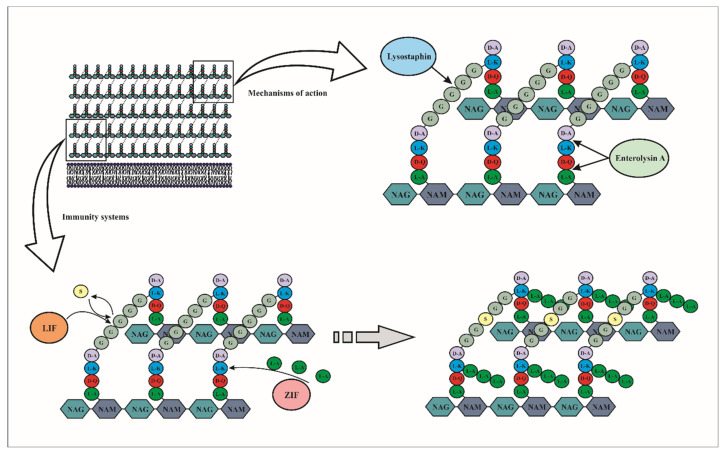
Mechanism of action and immunity of class III bacteriocins on the bacterial wall. These bacteriocins are endopeptidases that act on the bacterial cell walls. Lysostaphin cleaves specifically between the third and fourth glycine residues of the pentaglycine cross-bridge, while Enterolysin A cleaves interpeptide bonds inside peptidoglycan units between L-alanine and D-glutamic acid. Regarding the immunity, the Lif and Zif proteins add or modify the cross-bridged amino acids during the synthesis of peptidoglycan subunits in the cytoplasm. Therefore, the Zif protein increases the proportion of cross-bridges containing three L-alanines, while the Lif proteins are involved in the substitution of glycine by serine.

Depending on the nature of the lytic bacteriocin, the target may be either in the stem peptide, or in the interpeptide bridge, or both. Enterolysin A cleaves interpeptide bonds inside peptidoglycan units at two sites depending on the target strains. In *L. monocytogenes* NCTC 10884, *S. aureus* NCTC 4163 and *E. faecalis* No. 40, the cleavage occurs within the stem peptide between L-alanine and D-glutamic acid, whereas in *L. helveticus*, *L. casei*, *L. delbrueckii* ssp. *bulgaricus*, *P. pentosaceus*, *L. lactis* ssp. *lactis*, *L. lactis* ssp. *cremoris* and *L. helveticus* strains the cleavage occurs between the L-lysine of the stem peptide and the D-aspartic acid of the interpeptide bridge. It was reported that when enterolysin A cleaved within the peptide bridge between L-alanine and D-glutamic acid, the antimicrobial activity observed was significantly higher (Figure 6) [147].

The pentaglycine cross-bridge of the peptidoglycan represents the main target site of lysostaphin. For instance, in *S. aureus* and other staphylococcal species this bridge is constituted of five glycine (Gly) residues (Figure 6). Specifically, lysostaphin cleaves between the third and fourth glycine of the pentaglycine cross-bridge. It has been reported that the peptidoglycan of relatively lysostaphin-resistant staphylococcal species generally contains a greater amount of serine (Ser) than Gly [148].

However, some class III bacteriocins have a bacteriostatic instead of bactericidal mechanism of action, such as helveticin-M, a bacteriocin produced by *Lactobacillus crispatus*, which has an antimicrobial activity against *S. aureus*, *S. saprophyticus* and *Enterobacter cloacae*. In fact, the increase in cell membrane permeability caused by helveticin does not influence cytosolic enzymes, thus indicating a sublethal lesion [149]. The mode of action differs according to the bacterial species. It was reported that helveticin-M could disrupt the cell wall of Gram-positive bacteria and disrupt the outer membrane of Gram-negative bacteria, thus modifying the surface structure. Similarly, in Gram-negative bacteria, it also disrupted the inner membrane, resulting in leakage of intracellular ATP from the cells and depolarization of the membrane potential of target bacteria [149].

### 4.2. Immunity Mechanism of Class III Bacteriocins

Very few studies have been done on the auto-immunity of bacteriolysin producing strains to their own bacteriocin. Gargis et al. [150] studied the resistance to lysostaphin in *Staphylococcus simulans* biovar *staphylolyticus*. The lysostaphin resistance gene (lif) encodes a FemABX-like immunity protein, which is a non-ribosomal peptidyltransferase involved in the addition of cross-bridged amino acids during the synthesis of peptidoglycan subunits in the cytoplasm. In *S. simulans* bv. *staphylolyticus*, resistance to lysostaphin is due to the insertion of serines replacing certain glycines during peptidoglycan synthesis by the immunity protein. Indeed, it has been suggested that lysostaphin is unable to hydrolyze glycyl-serine or seryl-glycine bonds (Figure 6).

Other studies have shown that the immunity of producer cells to the bacteriocin zoocin A, a D-alanyl-L-alanine endopeptidase, was due to the presence of the zoocin A immunity factor (Zif), which also showed high degrees of similarity to members of the FemABX protein family (MurM and MurN). The authors reported that the presence of the Zif protein increased the proportion of crossbridges containing three l-alanines instead of two, resulting in reduced linkage of the recombinant target recognition domain of zoocin A to the peptidoglycan (Figure 6). In contrast to Lif, the Zif protein conferred resistance to zoocin A by lengthening the peptidoglycan cross-bridge rather than by causing an amino acid substitution [151].

Although there is a gene encoding an immunity protein in helveticin producing strains, the mechanism of immunity of these strains against their own bacteriocins has not been described [152,153]. 

## 5. Concluding Remarks and Future Prospects

LAB are known to produce many different antimicrobial substances such as bacteriocins, referred as LAB-bacteriocins. These molecules are of interest to the food industry, and can be used as biopreservative agents to replace chemical or heat treatments to produce foods with more natural preservatives, and rich in their organoleptic and nutritional properties [154]. Moreover, many LAB, particularly the genera *Lactococcus* and *Lactobacillus*, are considered safe to be used in animal feed and human food and health, and therefore they are included in the list of microorganisms with QPS (Qualified Presumption of Safety) status by the European Food Safety Authority (EFSA) in Europe [155]; or GRAS (Generally Recognized As Safe) status by the Food and Drug Administration (FDA) in the United States [156].

Accordingly, another application of LAB-bacteriocins could be in human medicine as an alternative to traditional antibiotics. The lack of discovery of new classes of antibiotics and the massive use of the traditional antibiotics has resulted in a worldwide antibiotic resistance issue. By 2050, it is predicted that antibiotic resistance will cause more than 10 million deaths per year, if no action is taken now. Apart from the cost in human lives, the financial cost of societal care will be more than USD 100 trillion worldwide [157]. Therefore, the use of LAB-bacteriocins in this context is completely justified. The majority of these molecules are safe [18] and also easy to be genetically modified in order to improve their activity. Advances in the chemical peptide synthesis and the availability of tools for expression of recombinant proteins will facilitate production of small bacteriocins with low post-translational modifications and extend their potential applications [158]. Moreover, bacteriocin-producing bacteria could be administrated to animals and humans in order to produce in situ their bacteriocins along the microbiota and, in this way prevent or help to alleviate bacterial infections [159,160].

Sometimes, the association of bacteriocins with other molecules could improve their activities. Zgheib et al. [161] reported that the combination of bacteriocins with nanoparticles could enhance their stability, their solubility, protect them from enzymatic damage, reduce their interactions with other drugs and improve their biopreservation. The understanding of the immunity mechanism is also a major issue. Indeed, it is important to know how the immunity proteins ensure protection against their associated bacteriocins in the producing strain. This would allow on the one hand a better understanding of the modes of action of bacteriocins and on the other hand it would prevent the emergence of potential pathogenic bacteria and emergence of resistance to bacteriocins.

## Figures and Tables

**Table 1:** Overview on the potential of LAB-bacteriocins applications.

Class	Bacteriocin	Producer Bacteria	Applications	Reference
Class I Lantibiotic	Nisin	*Lactococcus lactis* spp.	Food industry: natural preservative, inhibits most of the food-borne pathogens	[18]
			Human health: infection diseases, oral care, cancer therapy	[22]
	GP15cin	*Lactobacillus plantarum* GP15	Patented bacteriocin for control of unwanted bacteria in ethanol fermentation plants	[8]
	Salivaricin A2/B	*Streptococcus salivarius* K12	Active against Halitosis producing bacteria and *Streptococcus pyogenes*, which causes pharyngitis	[23,24]
	Lacticin 3147	*Lactococcus lactis* 3147	Inhibits the growth of many Gram-positive food-borne pathogens	[25]
			Treatment for bovine mastitis	[26]
Class I cyclic	Enterocin AS-48	*Enterococcus faecalis* subsp. *liquefaciens*	promising perspectives to be used as biopreservatives in food	[27]
			Active against uropathogenic enterococci	[28]
Class IIa Pediocin-like	Pediocin PA-1	*Pediococcus acidilactici* PAC1.0	Food industry: natural preservative	[29]
			Potential activity against fish pathogens	[30]
	ST4V	*Enterococcus mundtii* ST4SA	antibacterial and antiviral activity	[31]
			effective against middle ear infections	[32]
	Staphylococcin 188	*Staphylococcus aureu* AB188	Anti-enterococci and antiviral activity	[33]
	Enterocin CRL35	*Enterococcus faecium* CRL35	Anti-Gram-positive pathogens and anti-Gram-negative pathogens when hybridized to microcin V	[34]
	ST5H	*Enterococcus faecium* ST5Ha	Active against human food pathogens and virus	[35]
	Fermenticin HV6b	*Lactobacillus fermentum* HV6b MTCC	inhibits pathogens which causes vaginal infections in humans and has a spermicidal activity.	[36]
	Pediocin K2a2–3	*Pediococcus acidilactici* K2a2–3	Anti-cancer activity against HT-29 colon adenocarcinoma cells	[37]
Class IIb two-peptide	Durancin A5-11a/b	*Enterococcus durans* A5-11	Antifungical activity	[38]
	Plantaricin EF	*Lactobacillus plantarum* NCIMB8826-R	Anti-inflammatory effect and reduction of weight in obese mice	[39]
	Abp118	*Lactobacillus salivarius* UCC118	Enhancement of the bacteria probiotic effect and modulation of the mouse and pig intestinal microbiota	[40]
Class IIc Leaderless	Enterocin SL-5	*Enterococcus faecalis* SL-5	Active against inflammatory acne lesions caused by *Propionibacterium acnes*	[41]
Class III	Lysostaphin	*Staphylococcus simulans*	Active against *Staphylococcus aureus* and other pathogens of endometritis sows	[42,43]
